# Anti-Cyclic Citrullinated Peptide Antibodies and Severity of Interstitial Lung Disease in Women with Rheumatoid Arthritis

**DOI:** 10.1155/2015/151626

**Published:** 2015-05-19

**Authors:** Alberto Daniel Rocha-Muñoz, Manuel Ponce-Guarneros, Jorge Ivan Gamez-Nava, Eva Maria Olivas-Flores, Mayra Mejía, Pablo Juárez-Contreras, Erika Aurora Martínez-García, Esther Guadalupe Corona-Sánchez, Tania Marlen Rodríguez-Hernández, Mónica Vázquez-del Mercado, Mario Salazar-Páramo, Arnulfo Hernan Nava-Zavala, Ernesto German Cardona-Muñoz, Alfredo Celis, Laura González-Lopez

**Affiliations:** ^1^Centro Universitario de Tonalá (CUTONALA), Universidad de Guadalajara (U de G), 48525 Tonala, JAL, Mexico; ^2^Programa de Becarios de Investigación del Instituto Mexicano del Seguro Social (IMSS) y Programa de Doctorado en Farmacología, . Centro Universitario de Ciencias de la Salud (CUCS), U de G, 44340 Guadalajara, JAL, Mexico; ^3^Unidad de Investigación en Epidemiología Clínica y División de Investigación en Salud Unidad Medica de Alta Especialidad, Hospital de Especialidades, Centro Médico Nacional de Occidente (CMNO), Instituto Mexicano del Seguro Social (IMSS), 44340 Guadalajara, JAL, Mexico; ^4^Programa de Doctorado en Salud Pública, CUCS, U de G, 44340 Guadalajara, JAL, Mexico; ^5^Hospital General Regional-180, IMSS, 45650 Tlajomulco, JAL, Mexico; ^6^Instituto Nacional de Enfermedades Respiratorias “Dr. Ismael Cosío Villegas” (INER), 14080 Mexico City, DF, Mexico; ^7^Departamento de Medicina Interna-Reumatología y Departamento de Medicina Interna-Neumología, HGR-110, IMSS, 44700 Guadalajara, JAL, Mexico; ^8^Departamento de Fisiología, CUCS, U de G, 44340 Guadalajara, JAL, Mexico; ^9^Instituto de Investigacion en Reumatologia y del Sistema Musculoesqueletico, CUCS and Division de Medicina Interna, Servicio de Reumatologia OPD Hospital Civil Dr. Juan I Menchaca, U de G, 44340 Guadalajara, JAL, Mexico; ^10^Programa de Servicio Social en Investigación, Secretaria de Salud (SSA), 11570 Mexico City, DF, Mexico; ^11^Programa Internacional de Medicina, Universidad Autónoma de Guadalajara (UAG), 44670 Guadalajara, JAL, Mexico; ^12^Servicio de Medicina Interna, Departamento de Inmunología y Reumatología, Hospital General de Occidente, Secretaría de Salud Jalisco, 45170 Zapopan, JAL, Mexico; ^13^Dirección de la División de Disciplinas para el Desarrollo, Promoción y Preservación de la Salud, CUCS, U de G, 44340 Guadalajara, JAL, Mexico

## Abstract

*Objective*. To evaluate whether serum titers of second-generation anticyclic citrullinated peptide antibodies (anti-CCP2) are associated with the severity and extent of interstitial lung disease in rheumatoid arthritis (RA-ILD). *Methods*. In across-sectional study, 39 RA-ILD patients confirmed by high-resolution computed tomography (HRCT) were compared with 42 RA without lung involvement (RA only). Characteristics related to RA-ILD were assessed in all of the patients and serum anti-CCP2 titers quantified. *Results*. Higher anti-CCP2 titers were found in RA-ILD compared with RA only (medians 77.9 versus 30.2 U/mL, *P* < 0.001). In the logistic regression analysis after adjustment for age, disease duration (DD), smoke exposure, disease activity, functioning, erythrocyte sedimentation rate, and methotrexate (MTX) treatment duration, the characteristics associated with RA-ILD were higher anti-CCP2 titers (*P* = 0.003) and + RF (*P* = 0.002). In multivariate linear regression, the variables associated with severity of ground-glass score were anti-CCP2 titers (*P* = 0.02) and with fibrosis score DD (*P* = 0.01), anti-CCP2 titers (*P* < 0.001), and MTX treatment duration (*P* < 0.001). *Conclusions*. Anti-CCP2 antibodies are markers of severity and extent of RA-ILD in HRCT. Further longitudinal studies are required to identify if higher anti-CCP2 titers are associated with worst prognosis in RA-ILD.

## 1. Introduction

Rheumatoid arthritis (RA) is a chronic, systemic, inflammatory disease that involves synovial joints and other organs and extra-articular involvements associated with impairment in physical function, higher morbidity, and premature mortality [1, 2].

Interstitial lung disease (ILD) is an infrequent but extremely relevant extra-articular manifestation that decreases the patients' health-related quality of life (QOL) and life expectancy [2]. With the development of more accurate diagnostic methods, ILD has been reported with a prevalence of up to 61% in patients with RA [3]. ILD in RA is associated with around threefold the risk for mortality as compared with RA without this entity [4].

Some hypotheses concerning RA pathogenesis suggest that major susceptibility genes, particularly HLA-DR, shared epitopes that interact with smoking to trigger RA-specific responses to citrullinated proteins, signifying a clear relationship between smoking and the development of the immune response directed against citrullinated peptides [5]. One of the consequences of these reactions is the formation of anti-cyclic citrullinated peptide antibodies (anti-CCP), which are observed in around 55–69% of patients with RA [6]. These autoantibodies are highly specific markers for RA and are useful for predicting RA development and progression [7, 8], although the association between anti-CCP antibodies and extra-articular manifestations was not conclusive. Currently, there are few studies evaluating the association between anti-CCP autoantibodies and ILD in RA. Inui et al., on evaluating 18 patients with RA associated with ILD, did not find an association between anti-CCP and ILD [9]. On the other hand, Nikiphorou et al. recently demonstrated, in an abstract, their results of a multicenter study in which anti-CCP antibodies were strongly associated with ILD in RA [10]. Recently, Yin et al. identified ILD in 71 from among their 285 patients with RA, observing that positivity for second-generation anti-CCP (anti-CCP2) was associated with an increase in risk of ILD [11]. Kelly et al., from a multicenter study, identified 230 patients with proven ILD in RA. These authors identified that anti-CCP antibody titers comprised the most relevant factor associated with ILD in RA on univariate analysis, and this factor remained associated with ILD in the multivariate approach [12]. Reynisdottir et al., employing a different approach, analyzed the findings of high-resolution computed tomography (HRCT) in 70 patients with early, untreated RA who were positive for anticitrullinated proteins (ACPA-positive) compared with 35 patients with early, untreated ACPA-negative RA [13]. These authors identified that 63% of patients with ACPA-positive RA had abnormalities in HRCT compared with 37% of patients with ACPA-negative RA (*P* = 0.02) [13]. ILD is a major complication in RA, where prognosis is influenced by the presence of pulmonary active disease and severity of the lung involvement. Sathi et al. described that patients with RA with findings of more extensive lung disease on HRCT have a worse prognosis compared with patients with RA with limited ILD [14]. Currently, the extent and severity of ILD on HRCT and not merely the presence of ILD are considered as factors associated with the prognosis, leading to the development of tomographically validated scales to identify the severity of the lung involvement. Nonetheless, although the majority of studies have investigated the relationship of anti-CCP and ILD in RA, these studies have not evaluated whether there is an association of anti-CCP titers with the extent and severity of ILD on HRCT utilizing a validated scale.

To date, there is a lack of information on whether higher titers of these autoantibodies are related to clinical parameters for ILD severity, including the extent of lung damage assessed by validated tomographic scores. Therefore, our aim in this study was to examine the relationship between serum levels of anti-CCP2 and severity of the extent of ILD damage in patients with RA.

## 2. Materials and Methods

### 2.1. Study Design: Comparative Cross-Sectional Study

*Patients*. The study included patients with RA attending an outpatient rheumatology clinic in a secondary-care center (Hospital General Regional-110 of the Mexican Institute for Social Security (IMSS)) located in Guadalajara, JAL, Mexico. Patients were eligible if they met American College of Rheumatology 1987 classification criteria for RA [15] and were 18 years of age or older. Patients were not eligible if they had history of asthma or pulmonary tuberculosis, active respiratory infection, mental or psychiatric disorders, and any overlapped syndrome or if they exhibited an obstructive pattern during spirometry. Patients with criteria of MTX pneumonitis were excluded. From a cohort of 600 patients with RA, we identified 42 patients with RA with ILD data and these were compared with 39 patients with RA only selected consecutively from the same cohort and matched by gender and range of age.

In order to assess the ascertainment presence of ILD in RA, we performed a structured assessment of ILD based on the following strategy.

### 2.2. Definition and Ascertainment of ILD

Classification criteria for RA-ILD were based on the following three parameters:

- clinical symptoms, such as cough, phlegm, wheezing, bilateral inspiratory and expiratory crackles, and breathlessness,
- abnormalities in pulmonary function test (PFT) characterized by a decrease in forced vital capacity (FVC) <80% according to the predicted rate,
- radiographic evidence of ILD on HRCT, by means of bilateral outlying reticular opacities or honeycombing with or without activity for ground-glass pattern >5%.

Instead, criteria for inclusion of patients with RA without ILD (no RA-ILD) were based on the following three parameters:

- absence of clinical symptoms for lung involvement, such as cough, phlegm, wheezing, bilateral inspiratory and expiratory crackles, and breathlessness,
- PFT characterized by FVC ≥80% (predicted rate),
- no radiographic evidence of ILD on HRCT, by means of bilateral outlying reticular opacities or honeycombing ≤5% without activity for ground-glass pattern.

### 2.3. Clinical Assessment of Disease Characteristics of RA

A structured questionnaire was applied to patients to evaluate demographical, clinical, and therapeutic variables related with RA. The patients' synovial joints were examined for swelling and tenderness by a trained examiner. Disease activity was assessed employing the disease activity score in 28 joints (DAS28) [16], and functioning was assessed using the validated Spanish version for HAQ-Di [17]. Steinbrocker et al. [18] radiological stage and global functional status were also evaluated.

### 2.4. Cardiopulmonary Evaluation

Assessment of cardiopulmonary function included the following indices.

- The 6-minute walk test (6MWT) [19] was performed according to the American Thoracic Society (ATS) guidelines. The 6MWT measures the distance a patient can walk rapidly on a hard surface in a period of 6 minutes and is thought to reflect well a person's functional activity level for daily physical activities.
- The modified Borg scale [20] is a subjective scale that assesses the perception of dyspnea by the patient. This was performed immediately before and after the 6MWT, placing the degree of dyspnea on a scale of 0–100 mm, where 0 is none (no dyspnea) and 100 is the maximal dyspnea observed.
- The validated Mexican-Spanish version of the Saint George Respiratory Questionnaire (SGRQ) [21] consists of a self-administered questionnaire for measuring the impairment of patient-perceived health-related QOL in lung diseases in three domains, including symptoms, activity, and impact. Scores can range from 0 (no impairment) to 100 (worst impairment) for each domain; higher scores connote greater distress and, thus, worse health-related QOL. The questionnaire was administered and scored according to the instruction manual prior to the execution of 6MWT and PFT.

### 2.5. Screening with Pulmonary Function Tests (PFT)

A screening spirometry was performed with a SpiroPro, Sensormedics ver. 2.0, according to the recommendations published in 2005 by ATS and European Respiratory Society (ERS) [22]. The spirometries were performed to assess forced expiratory volume in 1 second (FEV_1_), forced vital capacity (FVC), and the FEV_1_/FVC ratio. Observed values were expressed as a percentage of the predicted value compared with individuals of similar gender, age, weight, and height. A restrictive pattern was defined as an FVC of <80% of that predicted in the absence of concomitant obstructive abnormality.

### 2.6. High-Resolution Computed Tomography (HRCT)

HRCT was performed using a single tomographer (4th generation equipment, Siemens SOMATOM AR.T. equipment). Following a standardized protocol, the HRCT was performed with the patient in prone position, using sections about 1-2 mm thick (at 10-mm intervals). Images were reconstructed with a high spatial algorithm and filmed using standard lung window settings (WL-700, [WW] 1000–1500 [HU]). HRCT scans were obtained at the suspended end-inspiratory volume with the patient in the supine position, and additional scans were obtained with the patient in the prone position, when necessary, to demonstrate the reversibility of high attenuation in dependent lung.

All images were evaluated independently and in random order by two observers (one experienced thoracic radiologist and an experienced pulmonologist) who were blinded to the clinical and pathological data. The final assessment was achieved by consensus with an adjudicator if there were disagreements in interpretation. Distribution patterns were visually assessed in three defined regions (upper, middle, or lower regions) of both lungs. The upper zone is from the superior aspect of the transverse aortic arch to the lung apices, the middle region from the top of the transverse aortic arch to the inferior pulmonary vein, and the lower zone from the inferior veins to the diaphragm. According to Kazarooni et al. [23], standardized sheet was used to tabulate the presence or absence of two features: (1) ground-glass opacity, defined as an area of increased attenuation, and (2) honeycombing, defined as subpleural clustered cystic air spaces with distinct walls of 3–25 mm in diameter, on a scale of 0–5 in the three lobes of both lungs as follows: 0: no alveolar disease; 1: ground-glass pattern involving <5% of the lobe; 2: involving >25%; 3: involving 25–45%; 4: involving 50–75%; 5: involving >75% of the lobe for the alveolar score and 0: nonfibrosis; 1: septal thickening without honeycombing; 2: honeycombing involving >25% of the lobe; 3: involving 25 to 49%; 4: involving 50–75%; 5: involving >75% of the lobe for the interstitial score. The sum of each pattern derives from the score of the three evaluated sections.

### 2.7. Anti-CCP and Other Laboratory Measurements

Erythrocyte sedimentation rate (ESR, mm/h) was measured using the Wintrobe method. Fasting sera were stored at −70°C until the determination of anti-CCP2 antibodies by enzyme-linked immunosorbent assay (ELISA), using a commercial sandwich ELISA kit (EUROIMMUN, Medizinische Labordiagnostika, AG, Lubeck, Germany), with cut-off values for seropositivity for anti-CCP2 of >20 U/mL.

### 2.8. Statistical Analysis

Quantitative variables were expressed as medians and ranges and qualitative variables as numbers and percentages. According to the distribution of anti-CCP2, we used nonparametric statistics. For comparisons of quantitative variables between patients with RA-ILD and RA only, we used Mann-Whitney *U* test, and for comparisons of qualitative variables between these groups, we utilized the chi-square test (or the Fisher exact test, if required). A correlation between anti-CCP2 titers with parameters of physical examination, disease activity indices, FVC, cardiopulmonary assessment, SGRQ domains, and HRCT scores was performed with Spearman's coefficient (*rho*). Significance was set at the 0.05 level. We built a multivariate logistic regression model with stepwise selection variables to identify risk factors for interstitial lung disease in RA. Thereafter, we performed a linear regression analysis in order to identify the variables associated with higher ground-glass and interstitial fibrosis scores in the HRCT. Those variables with a *P* value of <0.20 on univariate analysis or those with biologic plausibility to influence the development of RA-ILD were included in these multivariate models. All of the analyses were performed with SPSS ver. 8.0 statistical software.

### 2.9. Ethics

The study was approved by the Institutional Review Board of the Mexican Institute for Social Security (IMSS) of the participating hospital (approval number IMSS R-2010-1303-29), with all subjects providing written informed consent.

## 3. Results

**Figure 1:**
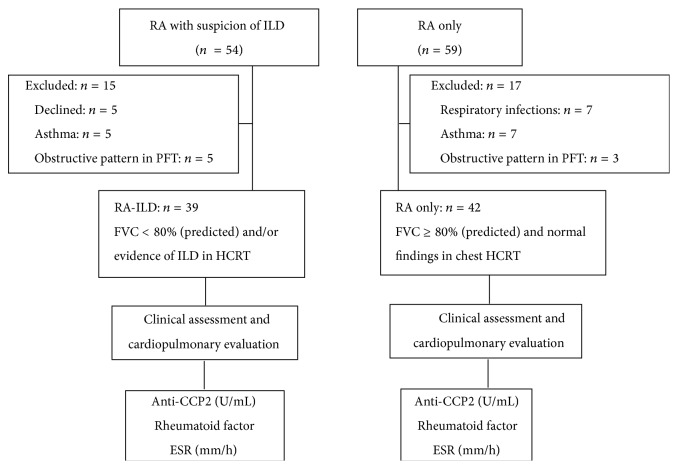
Study flow chart. RA: rheumatoid arthritis; RA-ILD: rheumatoid arthritis with interstitial lung disease; PFT: pulmonary function tests; HRCT: high-resolution computed tomography; FVC: forced vital capacity; anti-CCP: anti-cyclic citrullinated peptide antibodies; ESR: erythrocyte sedimentation rate.

Figure 1 presents the study's flow chart of the patients meeting the inclusion criteria. Of 54 RA patients with a suspicion of ILD, 15 were excluded from the study because they declined to participate (*n* = 5) or had exclusion criteria for the study (*n* = 10), whereas, of 59 RA patients without ILD, 17 were excluded because they met one of the exclusion criteria (see Figure 1).

Table 1 compares the characteristics of patients with RA-ILD (*n* = 39) versus RA only (*n* = 42). Patients with RA-ILD had higher scores for DAS28 (3.9 versus 2.5, *P* < 0.001) and HAQ-Di (0.8 versus 0.4, *P* < 0.001). Higher anti-CCP2 titers were found in patients with RA-ILD compared to RA only (77.9 versus 30.2 U/mL, *P* < 0.001); the frequency of positive rheumatoid factor (RF) was also higher in RA-ILD (97.4% versus 35.7%, *P* < 0.001), and levels of ESR were higher in ILD (32 versus 19.5, *P* < 0.001). A higher frequency of rheumatoid nodules history was found to be associated with the occurrence of RA-ILD (74.4 versus 14.7%, *P* < 0.001). Other findings associated with RA-ILD were higher frequency of higher MTX doses at the time of the study (*P* < 0.001), longer MTX duration (*P* = 0.002), and higher accumulated dose of MTX (*P* < 0.001). No statistical associations were observed between RA-ILD and age, the DD of RA, and smoking history.

**Figure 2:**
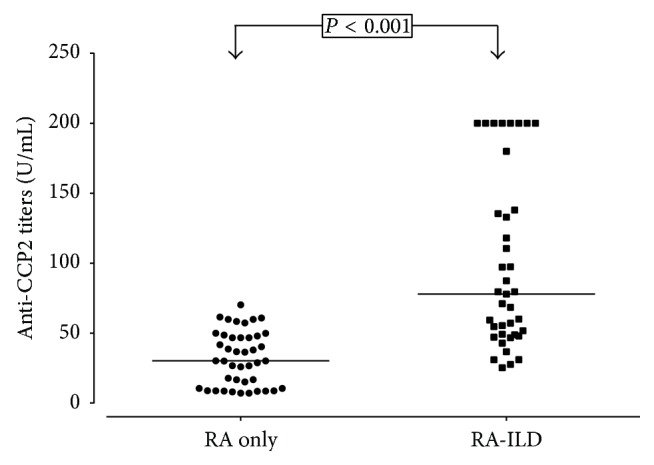
Anti-cyclic citrullinated peptide (anti-CCP2) titers in patients with rheumatoid arthritis without interstitial lung disease (RA-only), compared with patients with rheumatoid arthritis and interstitial lung disease (RA-ILD) group. The cut-off value of anti-CCP2 for positivity was 20 U/mL. Horizontal bars indicate the median. *P* values for the comparison of anti-CCP2 titers between groups were obtained by Mann-Whitney *U* test.

Figure 2 illustrates a comparison between anti-CCP2 titers in patients with RA only, compared with RA-ILD. Higher anti-CCP2 titers were observed in RA-ILD (77.9 versus 30.2, *P* < 0.001), whereas none of the patients with RA only had anti-CCP2 titers above 100 U/mL.

Table 2 compares the scores of cardiopulmonary scales and SGRQ between patients with RA only and patients with RA-ILD. Higher scores on the SGRQ and modified Borg scales following exercise were observed in RA-ILD (*P* < 0.001). Patients with RA-ILD had also lower distance in the 6MWT compared with RA only (310.0 versus 410.0, *P* < 0.001).

In data not shown in the tables, a correlation among anti-CCP2 titers was observed with DAS28 (rho = 0.420, *P* < 0.001), HAQ-Di (rho = 0.46, *P* < 0.001), SGRQ symptoms (rho = 0.547, *P* < 0.001), SGRQ activity (rho = 0.498, *P* < 0.001), SGRQ impact (rho = 0.518, *P* < 0.001), 6MWT (rho = −0.632, *P* < 0.001), pre-6MWT VAS modified Borg scale (rho = 0.637, *P* < 0.001), post-6MWT VAS modified Borg scale (rho = 0.619, *P* < 0.001), MTX treatment duration (rho = 0.293, *P* = 0.008), as well as FVC% (rho = −0.632, *P* < 0.001), and all the HRCT scores: ground-glass (rho = 0.566, *P* < 0.001) and interstitial fibrosis (rho = 0.70, *P* < 0.001).

In data that are not shown in tables, we performed a multivariable logistic regression analysis to identify variables associated with restrictive pattern in lung function tests. In the final model, the higher anti-CCP2 antibodies titers (OR 1.08 95% IC 1.02–1.14, *P* = 0.004) were associated with restrictive pattern in FVC, whereas factors that did not have statistical significance with FVC% were age, disease duration, RF, and years of treatment with MTX.

Table 3 shows the results of the multivariate logistic regression analysis to identify associated variables with RA-ILD. In the final model, after the adjustment for age, disease duration, smoke exposure, DAS28, HAQ-Di, ESR, and MTX treatment duration, two variables were associated with an increase of risk for RA-ILD: the higher anti-CCP2 antibodies titers (OR, 1.06; 95% CI, 1.02–1.10, *P* = 0.003) and positive RF (OR, 28.58; 95% CI 3.31–246.95, *P* = 0.002).

Table 4 presents the results of multiple linear regression analysis evaluating factors associated with higher severity of ILD according to the HCRT scores. After the adjustment for age, disease duration, DAS28, and MTX duration, we observed that the anti-CCP2 titers were significantly associated (*P* = 0.02) with higher severity of the extension in the ground-glass score. Similarly, after adjustment for age, DAS28 for the higher fibrosis score was significantly associated with higher disease duration (*P* = 0.01), anti-CCP2 titers (*P* < 0.001), and duration of treatment with MTX (*P* < 0.001).

## 4. Discussion

In the present study, anti-CCP2 titers were associated with the presence and severity of ILD in RA. These anti-CCP2 titers were correlated with impairment in several parameters for ILD severity, including the SGRQ, 6MWT, Borg scales, decrease in FVC%, and higher scores for ILD involvement and severity identified in the HRCT severity. An association between ILD and anti-CCP2 titers remained after adjustment for age, disease duration, and exposure to smoke, in the multivariate model and duration of treatment with MTX, whereas positive RF was also a factor associated with ILD. Additionally, we observed that the only factors that predicted in the multivariate linear regression the higher scores for fibrosis score were anti-CCP2 titers and longer MTX treatment duration; instead, higher fibrosis scores were inversely associated with disease duration.

Our results, regarding the association between anti-CCP2 and ILD, are similar to the findings by Nikiphorou et al. [10], who observed that anti-CCP2 titers are significantly higher in patients with RA who had ILD. On the other side, Inui et al. [9] did not observe an association between presence or levels of anti-CCP2 and ILD. One possible explanation for these differences between studies was that Inui et al. included only 18 patients with ILD associated with RA, and, therefore, because of the small sample evaluated, it is likely that this lack of differences can be explained by a type II error.

Recently, Yin et al. identified ILD in 71 from their 285 patients with RA, observing that positivity for second-generation anti-CCP (anti-CCP2) was associated with an increase in risk of ILD [11]. Kelly et al., from a multicenter study, identified 230 patients with proven ILD in RA. These authors identified that anti-CCP antibody titers were comprised of the most relevant factor associated with ILD in RA on univariate analysis and this factor remained associated with ILD in the multivariate approach [12]. Reynisdottir et al., using a different approach, analyzed the findings of HRCT in 70 patients with early untreated RA who were positive for anticitrullinated proteins (ACPA-positive) compared with 35 ACPA-negative RA [13]. These authors identified that 63% of ACPA-positive patients had abnormalities on HRCT, compared with 37% of ACPA-negative patients (*P* = 0.02) [13]. To date, to the best of our knowledge, there are no studies evaluating if the anti-CCP2 titers are associated with the extent and patterns of severity of lung involvement in ILD-RA. We reported that high titers of these autoantibodies are associated with a higher extent of lung involvement in our patients even after adjustment for other variables.

Reynisdottir et al. [13] observed increased staining for citrullinated proteins on bronchial biopsies obtained from patients with RA and positive anti-CCP. Rangel-Moreno et al. reported [24] higher levels of anti-CCP in serum and bronchoalveolar lavages in patients with RA, and these antibodies are increased in the patients with RA who had well-developed inducible bronchus-associated lymphoid tissue, suggesting that these antibodies are produced locally in the lungs. Citrullinated proteins in the lungs are currently considered as autoantigens that may trigger an immune response associated with the development of anti-CCP2 and other antibodies that may act as markers associated with the tissue damage [25]. In addition to anti-CCP2 levels, RF and elevated ESR were biomarkers associated with ILD in RA on univariate analysis. An association between positive RF and ILD has been previously identified by several studies [11–13]; our findings are consistent with their results, whereas our finding of an elevated ESR in patients with ILD in RA is inconsistent with the majority of the reported data. Yin et al. [11] did not observe differences in ESR between the group with ILD and the group without ILD in their study. On the other hand, Inui et al. [9] observed a nonsignificant trend for elevated ESR in patients with RA-ILD.

Instead, the association observed in the present study between MTX treatment and ILD is consistent with data reported in the literature. Roubille and Haraoui [26] examined evidence regarding the association between ILD and synthetic or biological DMARDs; these authors concluded, in their systematic review, that the incidence of MTX-associated pneumonitis has been estimated as ranging from 0.3 to 8% of patients with rheumatic disorders. Conway et al., in a meta-analysis, identified an increase in the risk of pneumonitis in patients receiving MTX [27]. Although in our study nearly all of the patients with RA received MTX at the time of the study, we were unable to identify if the patients not treated with this drug had lower risk for ILD.

Related to the association observed between anti-CCP2 and RA-ILD, ACPAs are specific for RA and correspond to a subset of RA that is distinct from RA ACPA-negative in terms of pathogenesis, disease prognosis, and response to therapy. This information about ACPAs suggests that the presence of autoimmunity to citrullinated peptides and the developmental may be initiated within the respiratory system. Not only are citrullinated proteins limited to synovial tissue, but they have also been identified at extra-articular sites in patients with RA. Bongartz et al. [28] found that citrullination is developed inside mononuclear cells in lung tissue in open-lung biopsy specimens from patients with RA-associated interstitial pneumonia. These authors also observed that, despite the high specificity of anti-CCP for RA, citrullination was also found in lung tissue from patients with idiopathic interstitial pneumonia. Posttranslational modification of citrullination is developed in an environment of inflammation. This protein citrullination is a phenomenon that is produced early in the disease course and that might be involved in the development of the disease. Zhu et al., in a meta-analysis, observed that ACPA-positive serum indicated a higher risk for ILD and interstitial pulmonary fibrosis (IPF) among patients with RA (OR, 4.679, 95% CI 2.071−10.572, *P* < 0.001) [29]. Giles et al. observed high serum ACPA titer associated with RA-ILD, after adjustment for confounding factors (age, sex, current or former smoking, and FR) [30].

A diagnosis of ILD in RA identifies a patient with higher risk for the worst prognosis considering that the median survival in RA-ILD is around 10 years shorter than that observed in the general population, lung disease being directly responsible of around 10 to 20% of all RA-associated mortalities [31]. Kelly et al. [12] identified that the subtype of usual interstitial pneumonia/overlap syndromes has around 3.9-fold risk for death in comparison with the subtype of nonspecific interstitial pneumonia/cryptogenic organizing pneumonia. An extensive disease had around 2-fold increase in the risk for death from any cause versus those limited diseases. Assayag et al. [31] identified in a systematic review that the extent of fibrosis and usual interstitial pneumonia is a predictor of mortality in RA-ILD.

Several studies observed that the positivity of anti-CCP2 and RF is associated with RA-ILD. Our study also observed in the multivariate logistic regression analysis that anti-CCP2 and RF were associated with RA-ILD. Our data are in agreement with the most recent studies. Yin et al. observed that positive rates of anti-CCP2 and RF in patients with RA-ILD were significantly higher than those of the patients with RA only [11]. However, Reynisdottir et al. found no significant difference in RF positivity in RA-ILD [13].

In our results, we are surprised that a shorter duration of disease was associated with the fibrosis score in the tomographic findings; several studies have associated the presence of ACPA-positive with the presence of pulmonary damage, mainly interstitial pulmonary and fibrosis pulmonary pattern; however, to our knowledge, no study has linked shorter duration of disease with RA-ILD and positive ACPA. Follow-up studies are required especially in patients with early RA, in which lung function comprises the value.

Our study possesses several limitations. This a cross-sectional design; therefore, it is unable to demonstrate the causality of any variable for the development of ILD in RA; however, our findings of the association between anti-CCP2 titers and the presence and severity of this complication are relevant for further studies in experimental models or longitudinal studies. In addition, none of our patients had a pulmonary biopsy. Thus, we have no information concerning the histological pattern exhibited by patients with ILD, and it would be interesting to evaluate whether these high anti-CCP2 titers may correlate with the histologic patterns in lung tissue involvement. On the other hand, to the best of our knowledge, there are no previous studies assessing whether the severity of ILD in RA is associated with higher titers of anti-CCP2. These findings of higher anti-CCP2 titers and ILD severity are not only limited to the HCRT score; they are also associated with other characteristics of impairment in ILD, such as decreased 6MWT, increases in the score for symptoms or impact in SGRQ, and decreases in FVC%. Another strength of the study was the utilization of a multivariate model to adjust the association of anti-CCP2 antibodies with ILD by other confounders; only two studies have previously used this statistical approach [10, 13], obtaining similar results to those observed in our study. In this respect, after adjustment for different factors, we observed that the relationship between the titers of these autoantibodies and the severity of ILD remained significant on multivariate analysis.

In conclusion, we identified that anti-CCP2 titers constitute an independent factor associated not only with the presence but also with the severity of RA-ILD; the relevance of these markers in patients with established ILD for future outcomes, such as progression of lung involvement and mortality, remains to be established.

## Figures and Tables

**Table 1:** Comparison in selected clinical variables between patients with RA and interstitial lung disease (RA-ILD) and patients with RA without interstitial lung disease (RA only).

Variable	RA patients groups		*P*
	RA-ILD *n* = 39	RA only *n* = 42	
Age, years	51.0 (36.0–72.0)	49.0 (24.0–73.0)	0.21
Smoking history, *n* (%)	9 (23.1)	13 (31.0)	0.46
Current smokers, *n* (%)	1 (2.6)	6 (14.3)	0.11
RA characteristics			
Disease duration, years	7.0 (1.0–35.0)	6.5 (0.75–25.0)	0.26
DAS28 (units)	3.9 (1.7–5.3)	2.5 (1.7–5.1)	<0.001
Inactive (<2.8)	13 (33.3)	29 (69.0)	0.002
Active (≥2.8)	26 (66.7)	13 (31.0)	
HAQ-Di (units)	0.8 (0.2–3.0)	0.4 (0.2–2.4)	<0.001
Impairment in HAQ-Di ≥0.6, *n* (%)	29 (74.4)	14 (33.3)	<0.001
Global functional status III-IV, *n* (%)	21 (44.7)	0 (0%)	—
Steinbrocker stage-hands, III or IV, *n* (%)	6 (12.8)	0 (0%)	—
Rheumatoid nodules history, *n* (%)	29 (74.4)	5 (14.7)	<0.001
ESR, mm/h	32.0 (14.0–62.0)	19.5 (8.0–45.0)	<0.001
Positive RF, *n* (%)	38 (97.4)	15 (35.7)	<0.001
Anti-CCP titers	77.9 (25.2–200.0)	30.2 (7.0–70.14)	<0.001
Positive anti-CCP	39 (100)	27 (64.3)	<0.001
DMARDs			
Methotrexate, *n* (%)	39 (100)	41 (97.6)	1.00
At the time of the study, mg/week	15.0 (10.0–22.5)	7.5 (0–12.5)	<0.001
MTX treatment duration, years	7.0 (2.5–30.0)	5.0 (0.8–13.0)	0.002
MTX accumulated doses, grams	6.8 (3.1–129.6)	1.1 (0–2.1)	<0.001
Azathioprine, *n* (%)	27 (69.2)	21 (50.0)	0.11
Chloroquine, *n* (%)	10 (25.6)	19 (45.2)	0.10
Corticosteroids utilization, *n* (%)	37 (94.9)	37 (88.1)	0.43

Qualitative variables were expressed in frequency (%); quantitative variables were expressed in medians (ranges); RA: rheumatoid arthritis; ILD: interstitial lung disease; ESR: erythrocyte sedimentation rate; VAS: visual analogue scale; HAQ-Di: Health Assessment Questionnaire-Disability Index; RF: rheumatoid factor; anti-CCP2: anti-cyclic citrullinated peptide antibodies (second generation); DMARDs: disease-modifying antirheumatic drugs. *P* values for comparisons between medians were computed with Mann-Whitney *U* test and for comparison between proportions were computed with chi-square (or Fisher exact test if applicable).

**Table 2:** Comparison of cardiopulmonary scales and Saint George Respiratory Questionnaire between patients with rheumatoid arthritis and interstitial lung disease (RA-ILD) and patients with RA only.

Variable	RA-ILD *n* = 39	RA only *n* = 42	*P*
Pulmonary symptoms			
Cough, *n* (%)^*^	31 (66.0)	0 (0%)	—
Phlegm, *n* (%)^*^	10 (21.3)	0 (0%)	—
Wheezing, *n* (%)^*^	3 (6.4)	0 (0%)	—
Bilateral inspiratory/expiratory crackles, *n* (%)^*^	33 (70.2)	0 (0%)	—
Breathlessness, *n* (%)^*^	19 (40.4)	0 (0%)	—
Cardiopulmonary scales			
6MWT, meters	310.0 (170.0–549.0)	410.0 (270.0–549.0)	<0.001
Pre-6MWT VAS modified Borg scale	1.0 (0–3.1)	0 (0–2.0)	—
Post-6MWT VAS modified Borg scale	2.0 (0.5–8.0)	1.0 (0–5.0)	<0.001
Development of dyspnea, *n* (%)	19 (23.8)	7 (16.7)	0.002
SGRQ, %			
Symptoms	14.0 (0–30.0)	3.0 (0–24.0)	<0.001
Activity	10.0 (0–38.0)	4.0 (0–27.0)	<0.001
Impact	10.0 (0–38.0)	3.5 (0–13.0)	<0.001
Total	13.0 (0–37.0)	5.0 (0–25.0)	<0.001
Lung function			
FVC (% of predicted)	71.0 (52.0–91.0)	86.0 (80.0–99.0)	<0.001
Restrictive patterns, *n* (%)^*^	32 (68.1)	0 (0%)	—

Qualitative variables were expressed in frequency (%); quantitative variables were expressed in medians (ranges); FVC: forced vital capacity; 6MWT: six-minute walk test; VAS: visual analogue scale; SGRQ: Saint George Respiratory Questionnaire. ^*^This variable is not accepted for evaluation by the program. *P* values were computed as follows: for quantitative variables with Mann-Whitney *U* test and for qualitative variables with chi-square (or Fisher exact test if required).

**Table 3:** Logistic regression analysis performed to assess the risk factors associated with the RA-ILD.

Criterion predictor	Method *Enter *			Method *Forward Stepwise *		
	OR	95% CI	*P*	OR	95% CI	*P*
Age, years	1.01	0.89–1.15	0.86	Not in the model	—	—
Disease duration >5 years	10.79	0.68–170.99	0.09	Not in the model	—	—
Smoke exposure	1.19	0.20–7.13	0.84	Not in the model	—	—
DAS28	0.29	0.03–1.48	0.12	Not in the model	—	—
HAQ-Di	1.17	0.12–11.16	0.89	Not in the model	—	—
ESR, mm/h	1.18	0.98–1.42	0.08	Not in the model		
Anti-CCP2 titers	1.05	1.01–1.10	0.01	1.06	1.02–1.10	0.003
+Rheumatoid factor	26.84	2.31–311.58	0.009	28.58	3.31–246.95	0.002
MTX treatment duration	1.60	1.00–2.56	0.05	Not in the model	—	—

DAS28: disease activity score; HAQ-Di: Health Assessment Questionnaire-Disability Index; ESR: erythrocyte sedimentation rate; MTX: methotrexate; anti-CCP2 titers: anti-cyclic citrullinated peptide antibodies titers (second generation); OR: odds ratios; 95% CI: 95% confidence interval. Variables were adjusted using logistic regression analysis. Dependent variable: presence or absence of interstitial lung disease. Covariates: age (quantitative), disease duration >5 years (qualitative), smoke exposure (qualitative), DAS28 (quantitative), HAQ-Di (quantitative), anti-CCP2 titers (quantitative), +rheumatoid factor (qualitative), and MTX duration in treatment (quantitative).

**Table 4:** Multiple linear regression analysis assessing the association of anti-CCP2 titers with the ground-glass and fibrosis scores observed in HRCT adjusting by selected variables.

Independent variables	HRCT			
	*Enter *		*Forward Stepwise *	
	*β*	*P* value	*β*	*P* value
Ground-glass score				
Age, years	0.026	0.85	—	Not in the model
Disease duration, years	0.068	0.90	—	Not in the model
DAS28	0.851	0.53	—	Not in the model
Anti-CCP2 titers	0.048	0.03	0.053	0.02
MTX treatment duration, years	−0.299	0.85	—	Not in the model
Fibrosis score				
Age, years	−0.069	0.19	—	Not in the model
Disease duration, years	−0.510	0.01	−0.506	0.01
DAS28	0.430	0.41	—	Not in the model
Anti-CCP2 titers	0.065	<0.001	0.070	<0.001
MTX treatment duration, years	0.879	<0.001	1.035	<0001

Anti-CCP2 titers: anti-cyclic citrullinated peptide antibodies titers (second generation); DAS28: disease activity score; HAQ-Di: Health Assessment Questionnaire; MTX: methotrexate. Dependent variables: first model: ground-glass score, second model: fibrosis score. Covariates included in this analysis were those quantitative variables that had statistical significance in the univariate analysis or were considered with biologic plausibility to explain the severity of ILD in HRCT.
